# Neuronal Variability during Handwriting: Lognormal Distribution

**DOI:** 10.1371/journal.pone.0034759

**Published:** 2012-04-13

**Authors:** Valery I. Rupasov, Mikhail A. Lebedev, Joseph S. Erlichman, Michael Linderman

**Affiliations:** 1 Department of Basic Research, Norconnect Inc., Ogdensburg, New York, United States of America; 2 Department of Neurobiology, Duke University, Durham, North Carolina, United States of America; 3 Department of Biology, St. Lawrence University, Canton, New York, United States of America; 4 Department of Neuroethics, Norconnect Inc., Ogdensburg, New York, United States of America; The University of Western Ontario, Canada

## Abstract

We examined time-dependent statistical properties of electromyographic (EMG) signals recorded from intrinsic hand muscles during handwriting. Our analysis showed that trial-to-trial neuronal variability of EMG signals is well described by the lognormal distribution clearly distinguished from the Gaussian (normal) distribution. This finding indicates that EMG formation cannot be described by a conventional model where the signal is normally distributed because it is composed by summation of many random sources. We found that the variability of temporal parameters of handwriting - handwriting duration and response time - is also well described by a lognormal distribution. Although, the exact mechanism of lognormal statistics remains an open question, the results obtained should significantly impact experimental research, theoretical modeling and bioengineering applications of motor networks. In particular, our results suggest that accounting for lognormal distribution of EMGs can improve biomimetic systems that strive to reproduce EMG signals in artificial actuators.

## Introduction

Neuronal variability is prominent even when behavioral actions are highly stereotypic. However, the nature, underlying mechanisms and statistical properties of this fundamental biological phenomenon are not well understood [1]–[4]. Here we studied statistical properties of EMG signals exhibited during handwriting. The methodology and technique of EMG recording during handwriting were described in our previous publication [5]. Handwriting consists of stereotyped hand movements that involve two basic motor components: firmly holding a pen by the fingers and moving the hand and the fingers to produce written text. In our experimental setup, a digitizing tablet (**Fig. S1**) is used to detect pen touch of the writing surface and pen movements. These records allow us to analyze individual handwriting trials where EMG records are precisely synchronized with pen movements. Such compatibility of time-dependent data is imperative for our trial-by-trial analysis because we examine EMG statistics for particular time instances measured from the handwriting onset.

## Methods

This study was approved by the Institutional Review Board of Human Participants Research of St. Lawrence University, Canton, NY and by the Institutional Review Board of Human Participants Research of Norconnect, Inc. No personal information was recorded during sessions and all data were analyzed anonymously. Written informed consent was obtained from the subjects prior to the EMG recording sessions.

We instructed eight subjects to write characters on a digitizing tablet while the surface EMGs of their intrinsic hand muscles were recorded by three electrode pairs. One pair sampled compound EMG activity from flexor pollicis brevis and abductor pollicis brevis. The second pair recorded from the first dorsal interesseus, and the third from the second and third dorsal interosseus muscles (**Fig. S2**). We found empirically that this electrode placement allows to capture major EMG modulations related to handwriting. Subjects wrote a single character more than 400 times. This took 10 blocks of 40 trials separated by 5 minute rest intervals.

**Figure 1:**
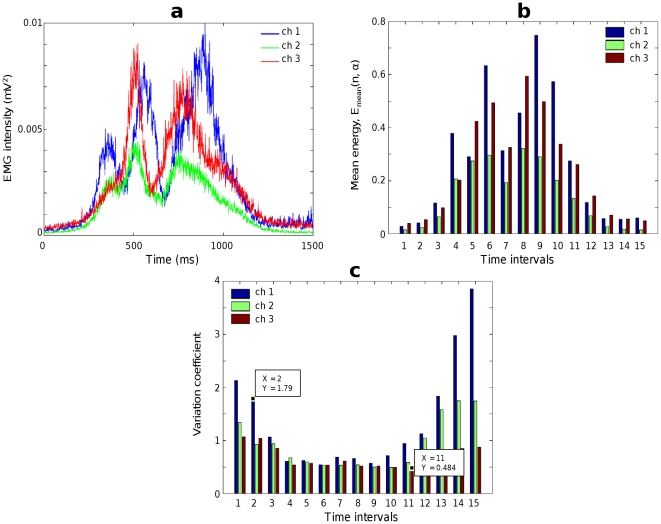
Statistical properties of raw EMG signals. (**a**) The intensities of raw EMG signals (digit “3”) averaged over 412 trials (templates); the point 500 ms on the time axis corresponds to the initial moment of time when a pen touches a paper. (**b**) The mean energy of the EMG signals in time intervals; the time interval 6 corresponds to the first 100 ms of the pen-on-paper period. (**c**) The variation coefficients of the distribution of dimensionless energies 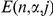.

We define a single handwriting trial as an epoch that starts 500 ms before the pen touches the tablet and ends 1000 ms after (**Fig. S3**). The amplitude of EMG signals, 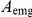, is squared to get the signal “intensity”, 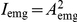. Trial-average intensities provide EMG templates that characterize the pattern of EMG activity during writing of particular character for each muscle (**Fig. 1a**).

To study time-dependent statistics of EMG signals, 1500-ms trials are subdivided into 15 time intervals, each with the duration of 100 ms (also commonly called bins). The signal “energy” is calculated for each of 15 intervals as the sum,
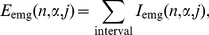
(1)where 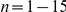, 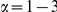, and 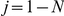 (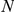 is the total number of trials), enumerate the time intervals, recording channels, and trials, respectively.

To obtain dimensionless variables for each interval, the energies 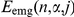 are normalized by dividing by the mean values, 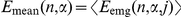. Here and hereafter 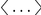 stands for averaging over trials,
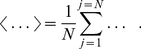
(2)Thus, in our analysis EMGs for each recording channel 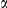 are characterized by dimensionless energies
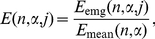
(3)whose time dependency is described by the discrete variable 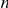 (interval number) and trial dependency is described by the variable 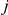 (trial number).

## Results

The typical time dependency of the mean energies 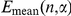 is shown in **Figure 1b**. The first five time intervals represent the epoch preceding the pen touch during which subjects first held the pen in the air and then approached the paper. The subject illustrated in **Figure 1b** wrote digit “3” with the mean duration of the pen-on-paper period (i.e., time during which the pen touched the paper) of 720 ms, which corresponds to time intervals 6 through 12, while the intervals 13–15 correspond to the time period after the subject lifted the pen from the paper.

The mean values of the normalized energies 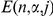 is equal to 1 by the definition (3), 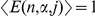, therefore the variation coefficients
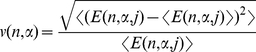
(4)which characterize the width of dispersion of the data distribution, are equal to the corresponding standard deviations. The distribution of each EMG signal had a wide dispersion: the variation coefficients were high for each time intervals and each channel (**Fig. 1c**), ranging for different time intervals from about 0.5 to almost 4. Therefore, EMGs during handwriting could not be adequately described in terms of mean values only, and a more detailed analysis of their statistical properties was required.

It should be noted that while the mean energies grew in the pen-on-paper period, the dispersion of the distribution was wide outside of this period, and substantially narrowed inside it. Thus, EMG patterns were more stable from trial to trial for stronger contractions. Such characteristics of the mean-energy and dispersion were observed for all subjects, handwritten characters, and muscles.

Although the duration of hand movement was variable from trial to trial, resulting in a misalignment of the liftoff periods, one could neglect this variability because the variation coefficient for hand movement duration was quite small, ranging for different subjects and characters from 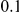 to 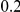.

**Figure 2:**
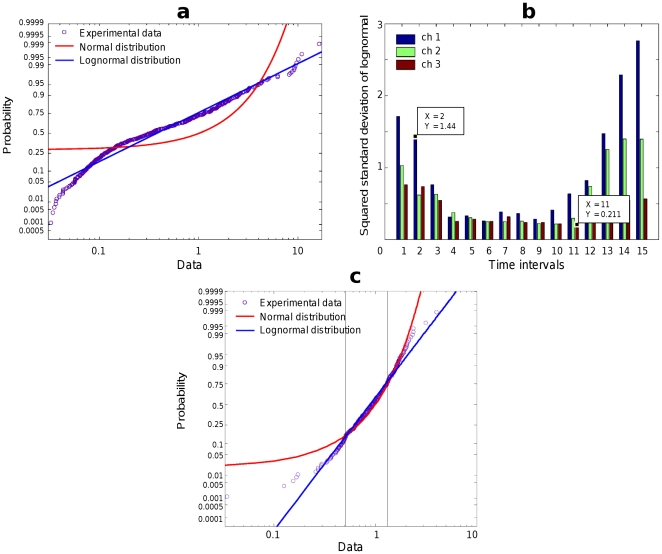
Probability plots. (**a**) Probability plot for experimental data for time interval 2, channel 1 (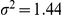) together with probability plots for theoretical normal and lognormal distributions. (**b**) The standard deviations for the lognormal distribution. (**c**) Probability plot for experimental data for time interval 11, channel 3 (theoretical 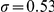 is shown by vertical lines).

Provided a sufficiently large number of trials were recorded, we could approximate a theoretical probability distribution for our data. In **Figure 2**, the probability plots for experimental data (with no trial selection) are shown together with probability plots for the theoretical normal and lognormal distributions. Axis scales are chosen to have a direct line for theoretical probability plot of the lognormal distribution. It is clearly seen that the lognormal distribution with the probability density function
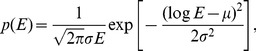
(5)where 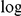 stands for natural logarithm, fits the experimental data very well.

Moreover, the lognormal distribution approximates the data distribution much better than the normal distribution (**Fig. 2a**). Parameters of the lognormal distribution - the “mean” 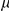 and the “standard deviation” 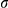 - are expressed in terms of the variation coefficient 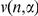 as follows:
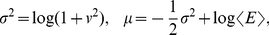
(6)that allowed us to compute the standard deviation 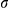 (**Fig. 2b**). Note, that the second term in the second equation vanishes because 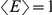 owing to the definition of dimensionless energies 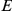 (Eq. 3). At sufficiently small 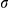 (strictly speaking, at 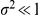), 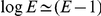, 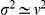, and the lognormal probability density function is approximately transformed into the normal probability density function,
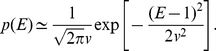
(7)Therefore, for time intervals and channels with relatively small 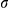, the lognormal and normal distributions approached each other, especially for the datapoints within one standard deviation from the mean (**Fig. 2c**).

**Figure 3:**
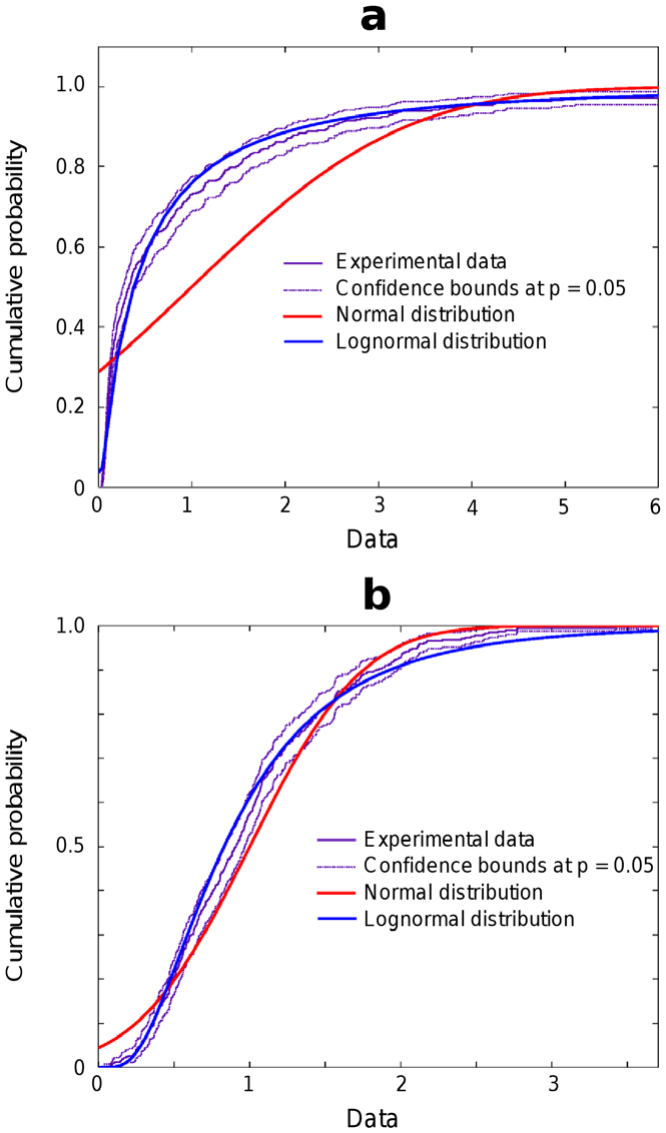
Cumulative distribution functions. (**a**) Plot of empirical cumulative distribution function (experimental data) for time interval 2, channel 1 (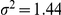) together with theoretical cumulative distribution function plots for normal and lognormal distributions. (**b**) Plot of empirical cumulative distribution function for time interval 11, channel 3 (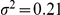). In both cases the confidence interval for experimental data is computed at p-value of 0.05.

The results were confirmed also by the comparison of the empirical cumulative distribution function [6] computed from experimental data with the theoretical lognormal cumulative distribution function. The theoretical curve for the lognormal distribution lies mainly inside the experimental confidence interval computed for p-value of 0.05 (**Fig. 3a**). On the contrary, the theoretical curve for the normal distribution lies well outside the confidence intervals at sufficiently for samples with large standard deviation (**Fig. 3a**) and approaches the confidence interval (**Fig. 3b**) for small 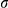.

**Figure 4:**
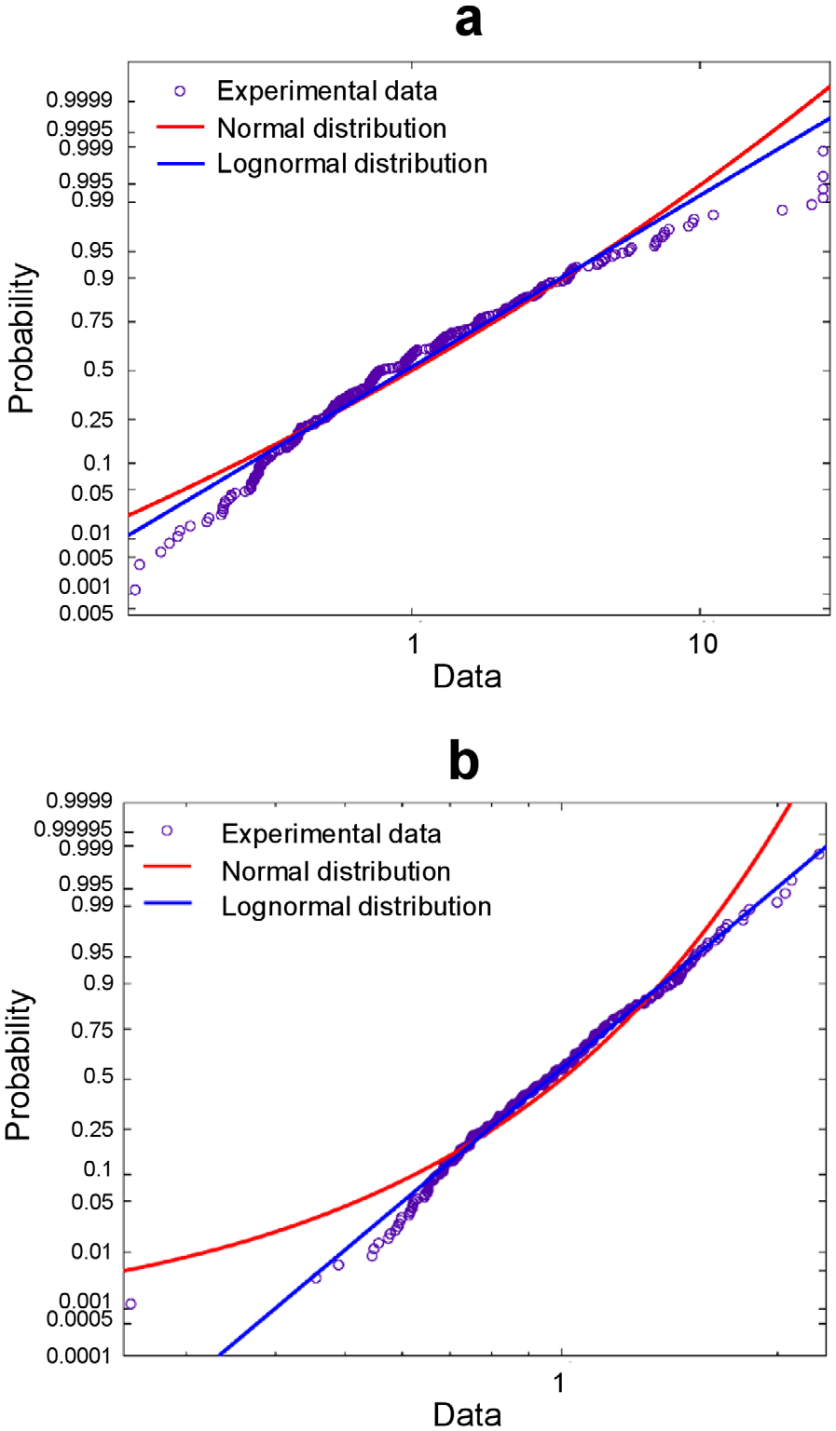
Distributions of time intervals. (**a**) Probability plot for the duration of the pen-on-paper period together with probability plots for theoretical normal and lognormal distributions. (**b**) Probability plot for time of reaction together with probability plots for theoretical normal and lognormal distributions. In both cases, the plot scales are chosen to have a direct line for the lognormal distribution.

Because of a low 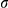, the distribution of handwriting durations on different trials was equally well approximated by both lognormal and normal distributions . Handwriting duration, 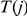, was computed as the duration of the pen-on-paper interval, and then normalized to the mean value, 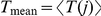, to get dimensionless variable 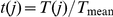. Since a typical variation coefficient was in the range of 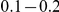 for all subjects and characters, the lognormal and normal probability density functions practically coincided (**Fig. 4a**).

Finally, we studied variability of the subject response to the computer “beep” that triggered the handwriting on each trial. The beep started 1 s after the subject lifted the pen from the paper on the previous trial. The response time, 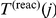, is computed as the time interval between the beep onset and the time when the pen touches the paper . After a normalization to the mean value 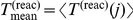 we derive a dimensionless variable 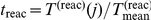 and study its distribution. In the case shown in **Figure 4b**, 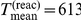 ms with the standard deviation of 179 ms.

It should be emphasized that EMG modulation started 200–300 ms before the pen touched the paper (see **Figs. 1a and 1b**), that is 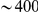 ms after the beep. Given that we did not ask the subjects to react as fast as they could, it is not surprising that the reaction time in our experiment was longer compared to the studies in which subjects received this instruction [7].

## Discussion

In conclusion, we found that the trial-to-trial variability of the EMG signals during handwriting is well described by the lognormal distribution for all time intervals and for all principal intrinsic hand muscles that we found to be active during handwriting. The standard deviation of the distribution 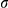 depends on the inter-trial time and muscle sampled (**Fig. 2b**). For sufficiently large 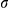, the lognormal distribution is well distinguished from the normal one, while for small 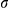 these distributions approach each other, as it would be expected, especially when the points are within one standard deviation from the mean [see Eqs. (6) and (7)]. Although the standard deviation of the lognormal distribution varies for different subjects and characters, we did not find any essential deviations from the lognormal distribution for any of 8 subjects who repeated about 400 trials for each of 3 characters.

We also found that trial-to-trial variability of handwriting temporal parameters was also well described by the lognormal distribution.

The lognormal distribution is often observed for variables which are a product of many independent random variables with arbitrary statistics [8]. It still remains to be studied if signal multiplication occurs in neuronal network that generated handwriting movements. Additionally, our EMG recordings from intrinsic hand muscles captured the activity of limited number of motor units: a few during moderate contractions and many during stronger muscle contractions. Because the lognormal distribution was most evident outside the pen-on-paper period, the results obtained may indicate that the trial-to-trial lognormal statistics is a characteristic feature of EMG signals comprising a few motor units. For stronger muscle contraction, when additional motor units were recruited in our experiments, the statistics shifted to the normal one in correspondence with the central limit theorem of the probability theory [6], which states that a sum of many independent random variables with arbitrary statistics is normally distributed.

It is noteworthy that lognormal functions also appear in theoretical models proposed and developed by Plamondon and coworkers for handwriting [9]–[12]. These models, based on quite general assumptions about the mechanism of muscle activity, well describe kinetics of fast human movements. In particular, the movement speed as a function of time is described by the difference of two lognormal functions with different sets of parameters.

Much interest was attracted recently [13], [14] to biomimetic interfaces that strive to reproduce biological motor functions, such as muscle activation. These interfaces use neural decoders that extract motor signals from brain and/or myoelectric activity. Modeling the statistics of bioelectrical signals involved, for example, using Bayesian models, is critical for the performance of such decoders. We suggest that using lognormal statistics may result in better accuracy of biomimetic interfaces.

## Supporting Information

Figure S1 **Experimental setup.**
(TIF)

Figure S2 **Schematics of sensor deposition at handwriting.**
(TIF)

Figure S3 **A typical pen-on-paper signal in one of trials.** The point of 500 msec corresponds to the initial moment of time when a pen touches a paper. EMG signals in all trials are synchronized with respect to this point.
(TIF)
