# Supplementary figures and images for: Neuronal Variability during Handwriting: Lognormal Distribution
Source: PLoS One. 2012 Apr 13;7(4):e34759. doi: 10.1371/journal.pone.0034759 (PMC3326033; doi:10.1371/journal.pone.0034759)

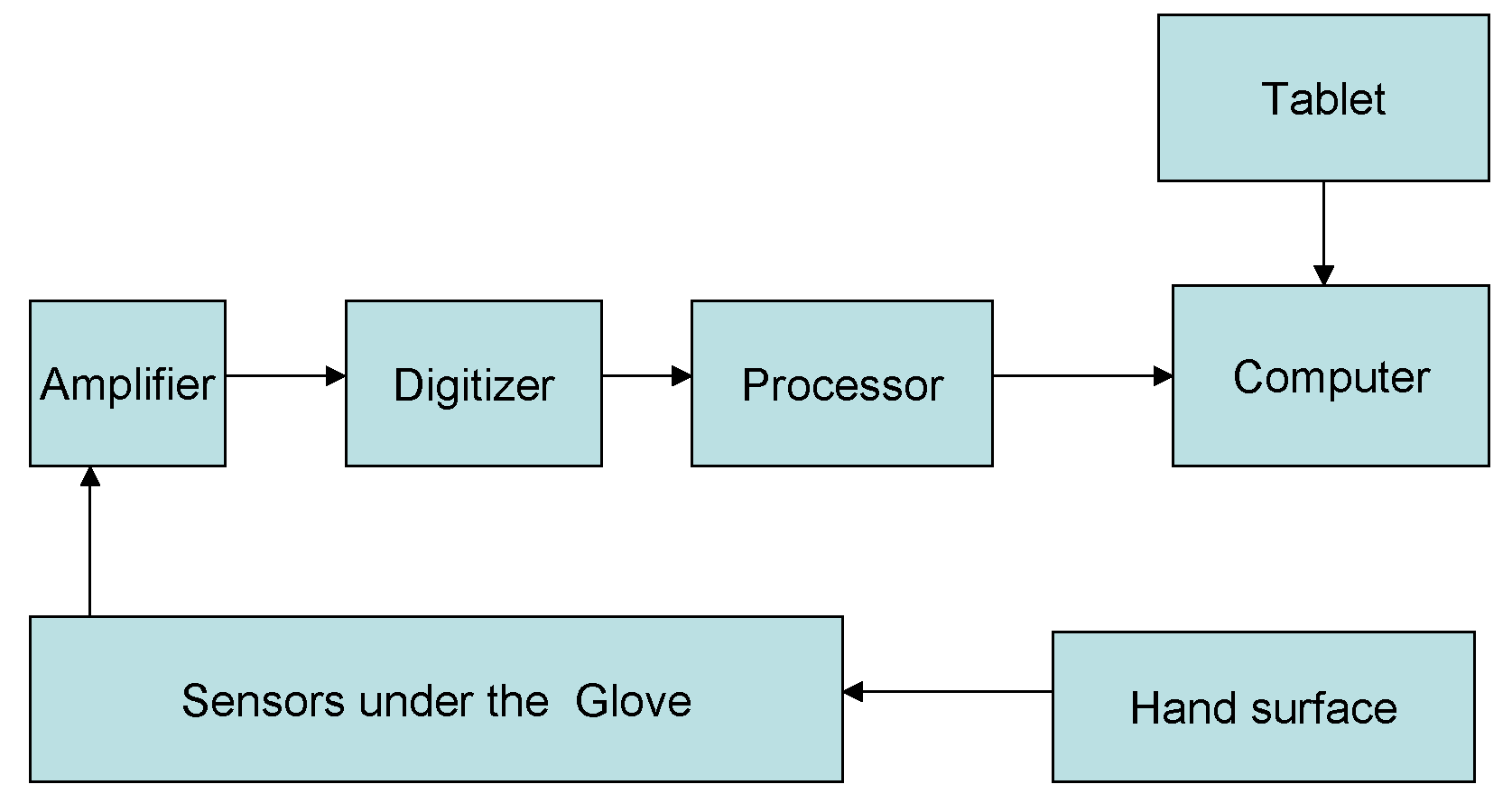

Supplement: Figure S1 — Experimental setup. (TIF) [file pone.0034759.s001.tif]

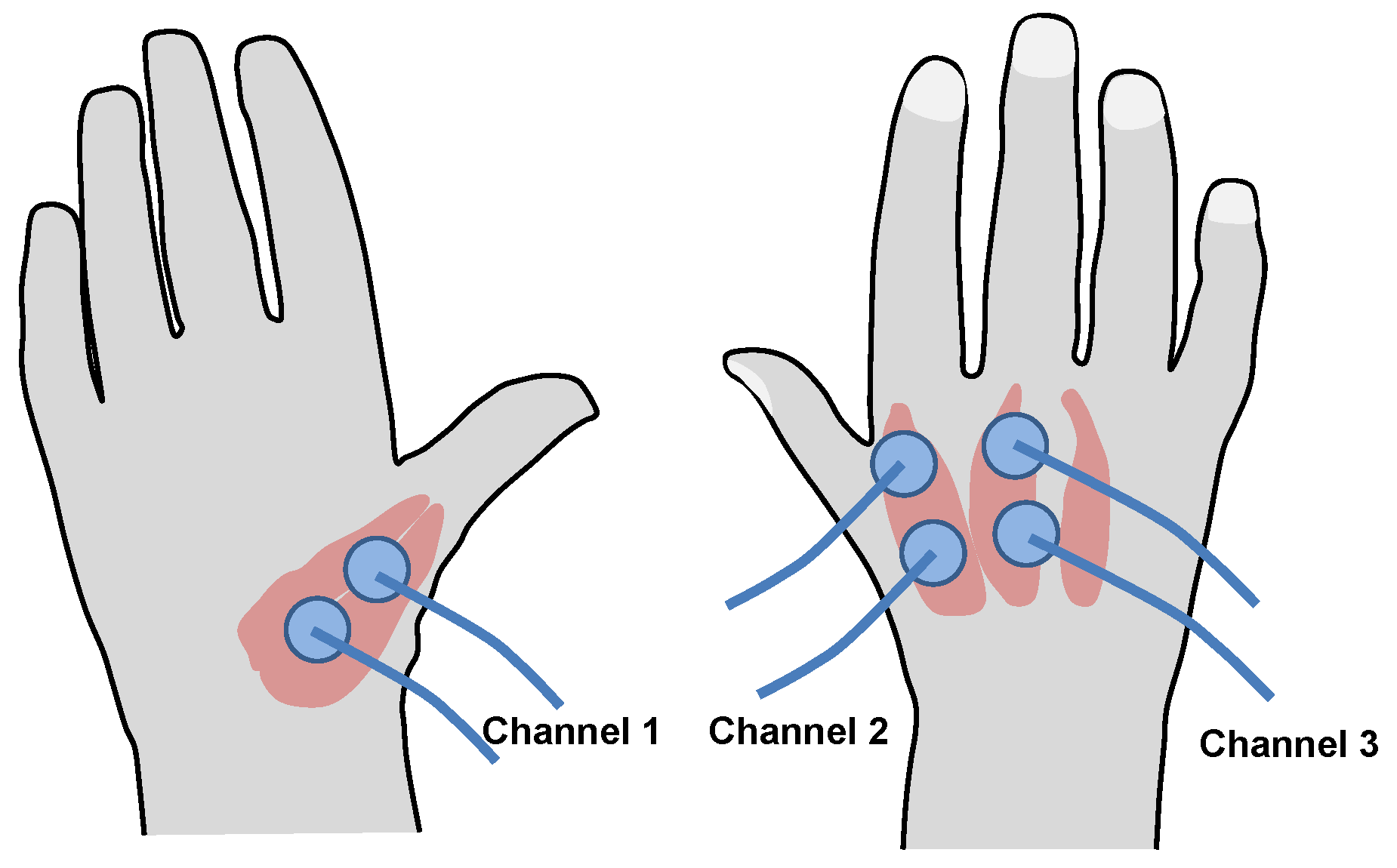

Supplement: Figure S2 — Schematics of sensor deposition at handwriting. (TIF) [file pone.0034759.s002.tif]

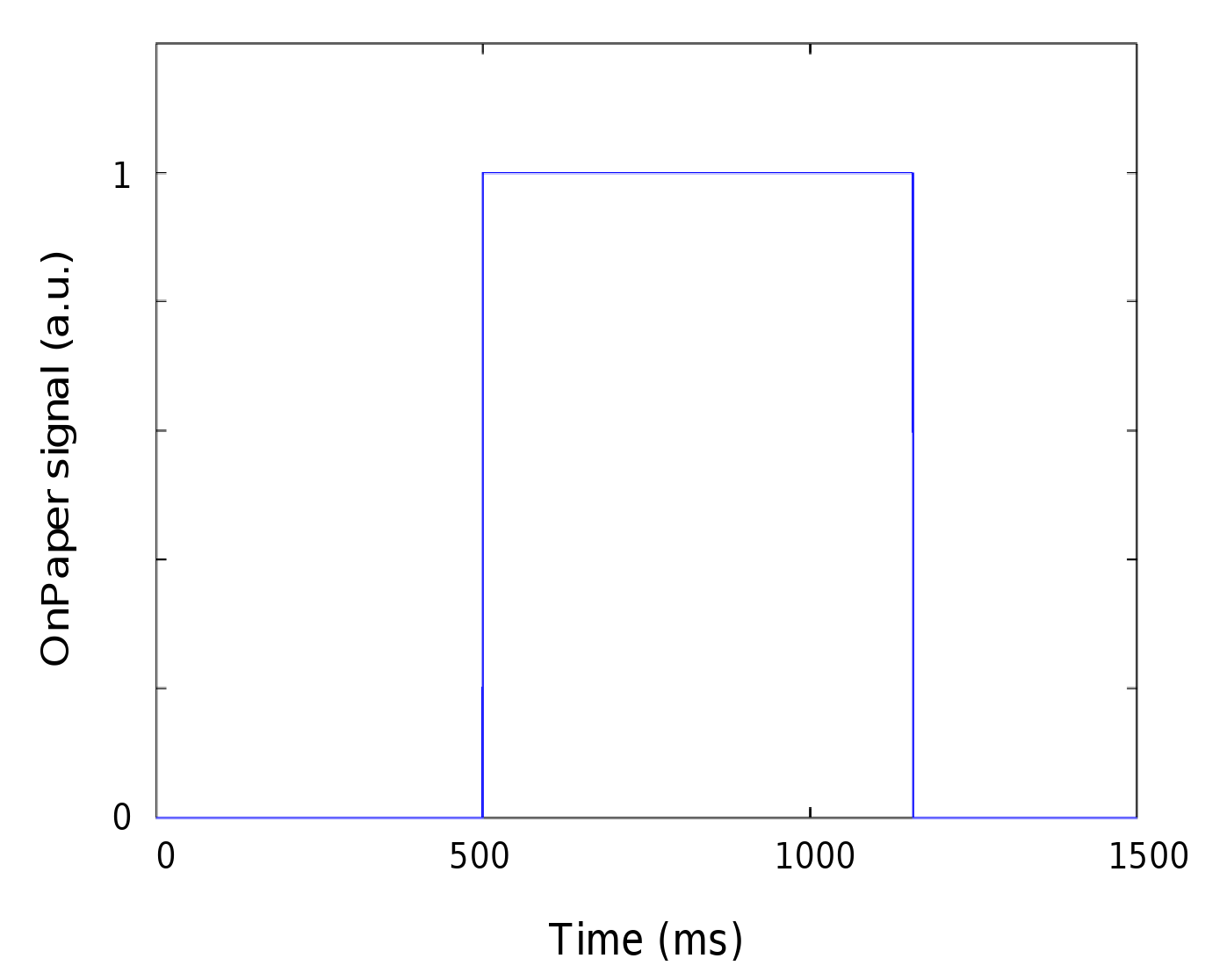

Supplement: Figure S3 — A typical pen-on-paper signal in one of trials. The point of 500 msec corresponds to the initial moment of time when a pen touches a paper. EMG signals in all trials are synchronized with respect to this point. (TIF) [file pone.0034759.s003.tif]
